# Interleukin 17 and peripheral IL-17-expressing T cells are negatively correlated with the overall survival of head and neck cancer patients

**DOI:** 10.18632/oncotarget.23934

**Published:** 2018-01-03

**Authors:** Meng-Hua Lee, Joseph Tung-Chieh Chang, Chun-Ta Liao, Ya-Shan Chen, Ming-Ling Kuo, Chia-Rui Shen

**Affiliations:** ^1^ Graduate Institute of Biomedical Sciences, College of Medicine, Chang Gung University, Taoyuan, Taiwan; ^2^ Department of Microbiology and Immunology, College of Medicine, Chang Gung University, Taoyuan, Taiwan; ^3^ Department and Graduate Institute of Medical Biotechnology and Laboratory Sciences, College of Medicine, Chang Gung University, Taoyuan, Taiwan; ^4^ School of Medicine, College of Medicine, Chang Gung University, Taoyuan, Taiwan; ^5^ Department of Radiation Oncology, Lin-Kuo Chang Gung Memorial Hospital, Taoyuan, Taiwan; ^6^ Department of Radiation Oncology, Xiamen Chang Gung Memorial Hospital, Xiamen, Fujian, China; ^7^ Department of Otorhinolaryngology, Head and Neck Surgery, Lin-Kuo Chang Gung Memorial Hospital and Chang Gung University, Taoyuan, Taiwan; ^8^ Division of Allergy, Asthma, and Rheumatology, Department of Pediatrics, Lin-Kuo Chang Gung Memorial Hospital, Taoyuan, Taiwan; ^9^ Department of Ophthalmology, Lin-Kuo Chang Gung Memorial Hospital, Taoyuan, Taiwan; ^10^ Chang Gung Immunology Consortium, Chang Gung Memorial Hospital and Chang Gung University, Taiwan

**Keywords:** head and neck cancer, PBMCs, interleukin-17, Th17 cells, prognosis

## Abstract

The presence and clinical significance of interleukin (IL)-17 and IL-17-expressing cells have recently been studied in several types of cancer, but their correlation to tumor development remains controversial. Additionally, the contribution of peripheral IL-17-expressing cells to head and neck cancer (HNC) progression is still poorly understood. We collected peripheral blood from healthy donors and HNC patients to isolate PBMCs. The percentages of IL-17-expressing cells and the production of inflammatory cytokines in PBMCs were measured to determine their association with clinical outcomes and overall survival in HNC. We evaluated the effect and potential mechanism of IL-17 on human oral squamous carcinomas *in vitro* using exogenous IL-17 stimulation. In comparison to healthy donors, the PBMCs of HNC patients have a significant accumulation of IL-17-expressing T cells and their frequencies were positively correlated with the disease stage. A significantly higher production of PBMC IL-17, TGF-β and IL-21 and plasma VEGF-A were found in HNC patients. Importantly, the 5-years overall survival of HNC patients with a higher percentage of IL-17-expressing cells is significantly decreased. Furthermore, the addition of IL-17 appeared to promote human oral squamous carcinoma cell proliferation via the production of IL-6 and VEGF-A. Our findings suggest that IL-17 has the potential to mediate pro-tumor immunity in the HNC tumor microenvironment. Enhanced IL-17-expressing cells, including Th17 and Tc17 cells, in the peripheral blood could be a significant predictor of a poor prognosis for HNC patients.

## INTRODUCTION

Head and neck cancer (HNC) is the sixth most common cancer worldwide and affects approximately 0.6 million patients per year [1]. Most head and neck cancers are squamous cell carcinomas that arise in the oral cavity, oropharynx, nasopharynx or larynx. Despite standard treatments, including surgery, chemotherapy, and radiotherapy, that improve the quality of life for HNC patients, patients often present with the advanced metastatic disease with frequent recurrences, resulting in a poor prognosis and a 5-year survival rate of only 50–60% [2, 3]. The incidence of HNC increases with age, especially in people in their 40s and 50s, and has a tremendous impact on family and societal life. Therefore, an understanding of the cellular and molecular changes that lead to the development of HNC could bring novel diagnostic and therapeutic procedures into clinical practice.

Alterations in host immune responses; the presence of immune cells, cytokines, and tumor infiltrates; and circulating T cells within the tumor microenvironment have a strong effect on HNC development [4, 5]. It has been established that tumors, including HNC, exert inflammatory effects from immune cells to influence cancer cell proliferation, migration, and survival [6, 7]. Studies in mouse models have shown that the premalignant stage of HNC development is associated with an increase in inflammatory Th1 and Th17 [8]. Furthermore, the inflammatory or inhibitory immune response in the tumor environment could be due to lymphocyte plasticity that is partially regulated by the cytokine milieu. Therefore, a better understanding of the immune status of cancer patients, particularly the distribution and regulation of pro-oncogenic and cancer-related inflammatory cytokines, will improve the clinical outcomes of HNC patients and provide new immunomodulatory approaches for HNC treatment. Interleukin (IL)-17 (IL-17A) is a pro-inflammatory cytokine that participates in both acute and chronic inflammatory responses and contributes to innate and adaptive immune responses in host defense and autoimmune disease [9, 10]. Th17 cells, a subtype of T helper cells, are the main producer of IL-17. However, it has been shown that CD8+ T cells (Tc17), γδ T cells and other innate immune cell populations, including macrophages and neutrophils, can also produce IL-17 [11–14]. Additionally, cytokines, such as TGF-β, IL-6, IL-1β, and IL-21, have been shown to be crucial for human Th17 cells differentiation *in vitro* [12, 15].

IL-17 and Th17 cells have recently been detected in various human and mice tumors, including gastric and ovarian cancer among other malignancies [16–20]. As an oncogenic mediator, it has been shown that IL-17 promotes tumor growth via angiogenesis and inflammation. IL-17 induces IL-6 production by tumor-infiltrating immune cells and tumor cells and activates the signal transducer and activator of transcription 3 (Stat3)-dependent pathway that subsequently enhances tumor cell growth [21]. By acting on malignant and tumor stromal cells, IL-17 induces a wide range of pro-angiogenic factors, such as vascular endothelial growth factor (VEGF), prostaglandin E1 and prostaglandin E2, to mediate tumor metastasis [18, 22]. In addition, IL-17 can also upregulate chemokine expression in the tumor environment to facilitate regulatory T cell (Treg) and myeloid-derived suppressor cell (MDSC) infiltration to suppress the anti-tumor immune response [23–25].

Even though several studies have focused on the proportion of Th17 cells in distinct human cancers, the prevalence and clinical significance of IL-17-expressing cells in HNC patients have not yet been well examined. Thus, the focus of our study is on the impact of the IL-17 on HNC pathogenesis and tumor immunity by evaluating the relevance of peripheral IL-17-expressing T cells to clinical parameters. In the current study, we characterized the phenotype, cytokine profile and clinical significance of PBMCs in HNC patients and revealed that IL-17-expressing cell populations in the peripheral blood of HNC patients were increased compared to healthy controls. In addition, we also examined the clinical significance of the increase of peripheral IL-17-expressing cells in HNC patients. We found that the higher prevalence of IL-17 and IL-17-expressing T cells was positively correlated with disease progression and a poor overall survival. Furthermore, we demonstrated that the downstream mechanism that works downstream of IL-17 to modulate pro-carcinogenic effects on human oral squamous carcinoma (OSCC) cells was the stimulation the production of IL-6 and VEGF-A. Thus, our study shows that IL-17 and peripheral IL-17-expressing T cells have a substantial impact on pro-tumor immunity and tumor pathogenesis in patients with HNC and could serve as HNC prognosis predictors.

## RESULTS

### The induction of peripheral IL-17-expressing cells is associated with tumor progression in head and neck cancer

**Figure 1:**
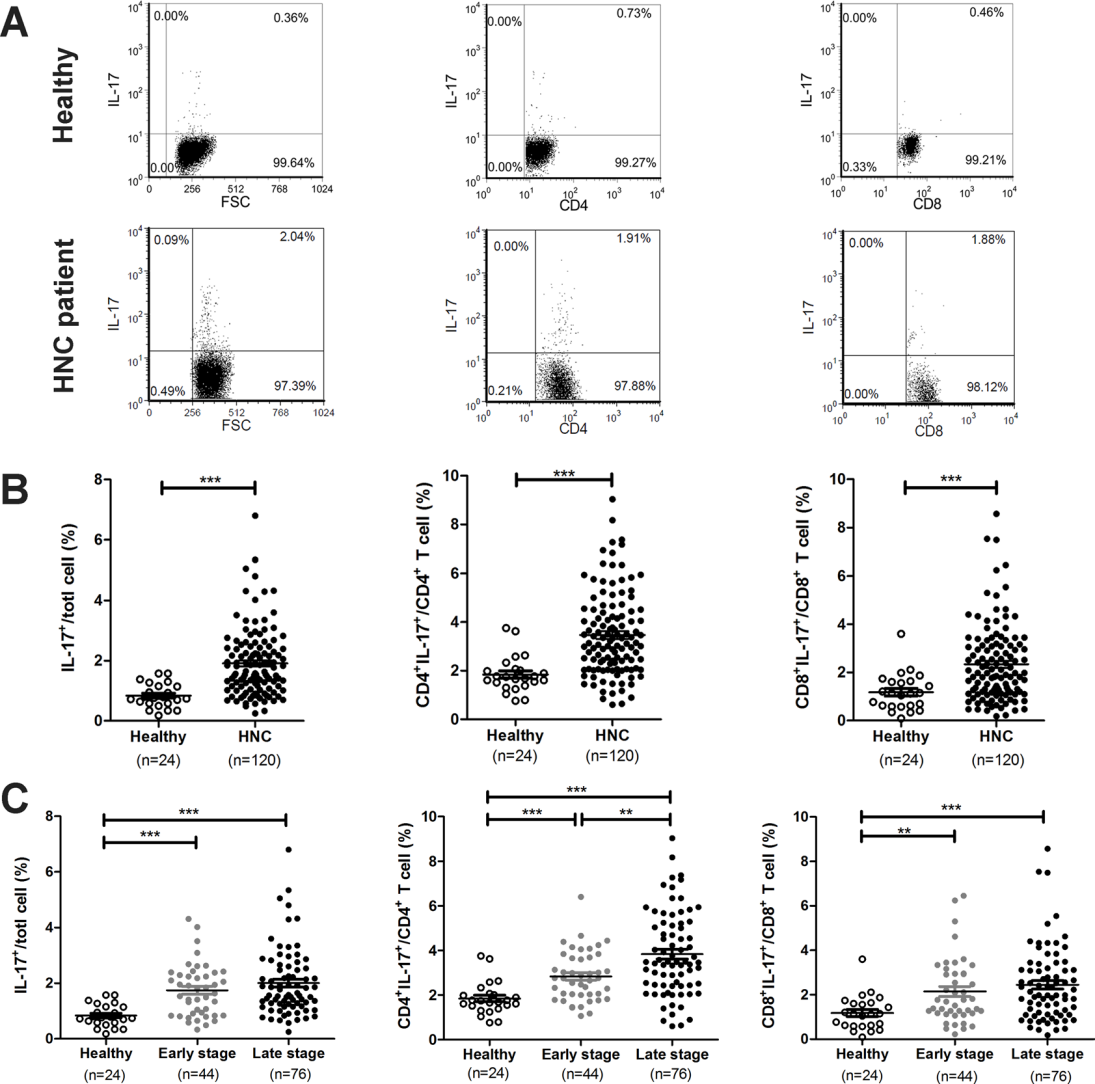
The frequency of IL-17-expressing T cells increases in the peripheral blood of patients with HNC Isolated PBMCs from healthy donors and patients with HNC and the percentage of IL-17-expressing cells, Th17 and Tc17, was evaluated by flow cytometric analysis. (**A**) Representative plots for identification of IL-17 producing cells in PBMC, CD4+ or CD8+ populations. (**B**–**C**) Analysis of the frequency of IL-17 producing cells from the PBMCs of healthy donors in comparison to HNC patients with tumors at different stages. Each dot represents an individual sample.

To examine whether peripheral IL-17-expressing cells are associated with HNC tumor progression, we first analyzed the frequency of IL-17^+^ cells, including the population of T cells in the peripheral blood, of patients with HNC. One hundred and twenty HNC patients were included in this analysis, and their clinical characteristics are summarized in Table 1. Figure 1A presents the representative flow cytometry data used for analyzing the population of IL-17-expressing cells in PBMCs. The proportion of peripheral IL-17^+^ cells in HNC patients was significantly increased compared to healthy controls (HNC: 1.91 ± 0.10% vs. healthy controls: 0.84 ± 0.08%, *P* < 0.001, Figure 1B). It is known that T cells are a major source of IL-17 production in many inflammatory diseases [12]; thus, we next assessed the phenotype of CD4^+^IL-17^+^ (Th17) and CD8^+^IL-17^+^ (Tc17) cells in the PBMCs of HNC patients. The percentage of peripheral Th17 cells (HNC: 3.47 ± 0.16% vs. healthy controls: 1.85 ± 0.15%, *P* < 0.001) and Tc17 cells (HNC: 2.34 ± 0.15% vs. healthy controls: 1.18 ± 0.16%, *P* < 0.001) in patients with HNC were significantly higher than those in healthy controls (Figure 1B). Furthermore, it appeared that the peripheral IL-17^+^, Th17, and Tc17 cells were all increased in both early and advanced stage HNC patients. In fact, the frequency of Th17 cells was significantly increased in patients with advanced stage HNC compared to patients with early stage HNC and healthy controls (Figure 1C), indicating a possible association between the presence of Th17 cells and tumor progression.

**Table 1:** Clinicopathological characteristics of 120 patients with head and neck cancer

Characteristics	No. (%)
Age (years at disease onset)	
50	58 (48.3)
50	62 (51.7)
Gender	
Male	99 (82.5)
Female	21 (17.5)
T classification^a,b^	
T1	14 (11.7)
T2	61 (50.8)
T3	14 (11.7)
T4	31 (25.8)
N classification^a,b^	
N0	45 (37.5)
N1	30 (25.0)
N2	30 (25.0)
N3	15 (12.5)
Overall stage^a,b^	
I	8 (6.7)
II	36 (30.0)
III	22 (18.3)
IV	54 (45.0)
Sub-site	
Oral Cavity	59 (49.2)
Nasopharynx	43 (35.8)
Oral pharynx, larynx, and hypopharynx	18 (15.0)

^a^Pathological T/N classification and stage for oral cavity cancer.

^b^Clinical T/N classification and stage for oral pharyngeal, laryngeal, and hypopharyngeal cancer.

To confirm the clinical relevance of the increase in Th17 and/or Tc17 cells in the peripheral blood of HNC patients, we analyzed the correlation between these cells and age, gender and the clinicopathological status, including overall stage (I+II and III+IV), tumor (T) factors, and lymph node metastasis (N) factors. As shown in Supplementary Table 1, there were no significant differences in the frequencies of peripheral Th17 and Tc17 cells regarding age, gender, and lymphatic metastasis. However, an elevated percentage of Th17 and Tc17 cells was found in patients with extensive tumor invasion depth (T3+T4) compared to patients with T1+T2, although only Th17 cells in advanced stage (III+IV) were significantly higher than those at an early stage (I+II). These results indicated that the increase in IL-17-expressing cells in peripheral blood, particularly Th17, was associated with tumor progression in HNC.

Alterations in cytokines production can lead to phenotypic changes in immune cells that contribute to tumor immune-surveillance. We next examined the expression of IL-17 and Th17-associated inflammatory cytokines, including TGF-β, IL-6, and IL-21, in PBMCs from HNC patients. The PBMCs from HNC patients (*n* = 62) produced relatively more IL-17 and significantly higher levels of IL-21 and TGF-β, but lower levels of IL-6 and IL-1β compared to healthy controls (*n* = 11). Similarly, PBMCs from patients with advanced HNC produced higher levels of IL-17 compared to patients with early stage HNC and healthy controls, although these differences were not statistically significant (Table 2). Moreover, we found no significant increase in the production of other inflammatory cytokines (TNF and IFN-γ) and chemokines (MCP-1, IP-10, and MIG) from HNC patients compared to healthy controls (Supplementary Table 2). We also found that these cytokines are not expressed in plasma and serum samples from HNC patients (data not shown). Therefore, our results suggested that PBMCs from HNC patients possess the ability to produce a high level of IL-17 and the Th17-associated cytokines IL-21 and TGF-β, which may reflect the disease severity.

**Table 2:** Levels of IL-17 associated cytokines produced from PBMC contributing to HNC clinicopathologic status

	IL-17	IL-21	TGF-β	IL-6	IL-1β
Healthy donor	125.2 ± 22.19	31.98 ± 10.11	1873 ± 242.3	2042 ± 427.9	1198 ± 283.5
HNC	279.5 ± 33.72	86.84 ± 7.530	3368 ± 255.9^*^	659.7 ± 143.9^***^	525.5 ± 131.2^*^
Early stage (I + II)	197.3 ± 46.79	108 ± 17.28^**^	3124 ± 459.3	832.2 ± 268.5^*^	857.5 ± 307.6
Late stage (III + IV)	327.9 ± 44.58^*^	74.34 ± 5.63^***^	3513 ± 305.9^**^	558 ± 165.9^***^	319.1 ± 92.56^***^

^a^Healthy donor *n* *=* 11, Early stage *n =* 23 , Advanced stage *n =* 39.

^b^Data were presented as means ± SEM (pg/mL).

^c^*P* value reflects t test analysis of cytokine values from HNC patients compared with healthy donors.

^*^P < 0.05, ^**^P < 0.01, ^***^P < 0.001.

### The increase in tumor-elevated IL-17 in peripheral blood predicts poor prognosis of HNC

**Figure 2:**
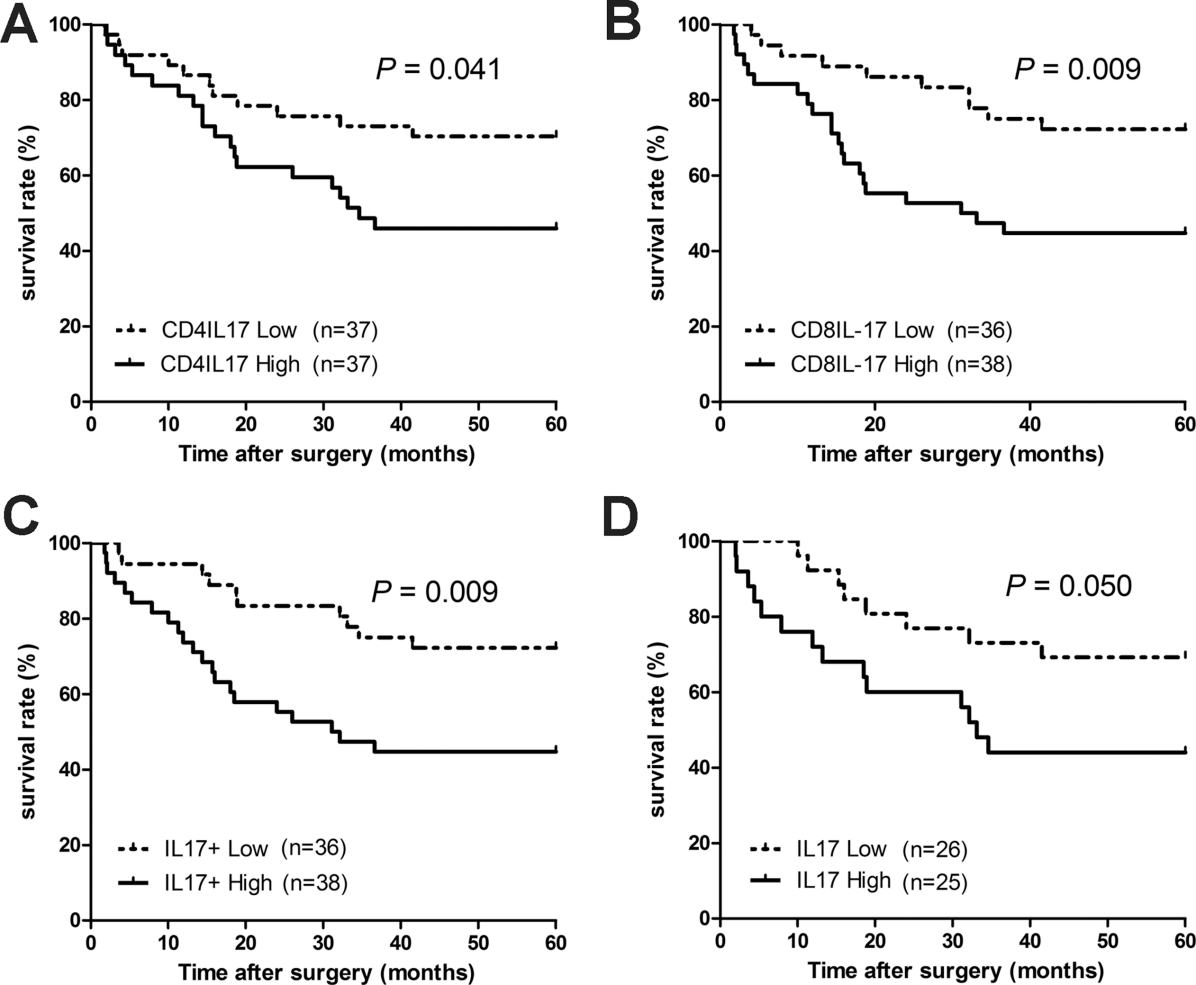
The expression of peripheral blood IL-17-producing T cells is negatively associated with 5-year survival for HNC patients We correlated expression of peripheral blood IL-17-producing T cells in HNC patients with their survival rates after surgery. 72 HNC patients were divided into 2 groups based on their median levels (%) of IL-17 producing cells. The 5-year overall survival rates for HNC patients with a high and low level of (**A**) Th17, (**B**) Tc17, and (**C**) IL-17^+^ cells in their PBMCs and (**D**) IL-17 from PBMC culture supernatant. The survival rates were determined using the Kaplan-Meier method (log-rank test).

Patients with lung cancer [26], hepatocellular carcinoma [27] and colorectal carcinoma [18] have high expression of intratumoral IL-17, which correlates with tumor prognosis. We, therefore, evaluated the clinical relevance of IL-17 expression in PBMCs from HNC patients by analyzing the impact of peripheral IL-17-expressing cells on overall survival for patients with HNC. In our study, 72 HNC patients were divided into two groups based on the median percentage levels of IL-17-expressing cells in the PBMCs (IL-17^+^: 1.50%, Th17: 3.015% and Tc17: 1.81%). As shown in Figure 2, a correlation analysis was performed with the 5-year survival rate and the high and low levels of peripheral IL-17^+^, Th17, and Tc17 of the HNC patients. Patients with a high percentage of IL-17-expressing cells in their PBMCs have a significantly lower 5-year overall survival rate compared to patients with a low percentage of IL-17-expressing cells (Figure 2A, 44.7% vs. 72.2%, hazard ratio (HR) = 2.591, 95% confidence interval (95% CI) = 1.27–5.28, *P =* 0.009). The 5-year overall survival rate of patients in the high Th17 group was lower than that in the low Th17 group (Figure 2B, 45.95% vs. 70.27%, HR = 2.095, 95% CI = 1.03–4.25, *P* = 0.041), and patients in the high Tc17 group have a significantly higher death hazard rate compared with those in the low Tc17 group (Figure 2C, 44.7% vs. 72.2%, HR = 2.568, 95% CI = 1.26–5.23, *P* = 0.009). In addition, patients were divided into two groups based on the median production values of IL-17 (160.9 pg/mL) from their PBMCs. We found that patients in the high IL-17 production group have a relatively lower overall survival rate compared with those in the low IL-17 production group (Figure 2D, 44.0% vs. 69.2%, HR = 2.331, 95% CI = 1.00–5.47, *P* = 0.050). The statistical analysis of overall survival demonstrated a negative correlation between overall survival and the accumulated peripheral IL-17-expressing cells in HNC. The univariate analysis demonstrated that high frequencies of IL-17-expressing cells (including Th17, Tc17, and IL-17+ cells), tumor (T) factors, lymph node metastasis (N), and TNM staging were significantly associated with poor overall survival in HNC, though all covariates did not reach statistical significance in the subsequent multivariate analysis (Supplementary Table 3).

### IL-17 activates IL-6 production in oral squamous carcinoma cells to promote tumor cells growth

**Figure 3:**
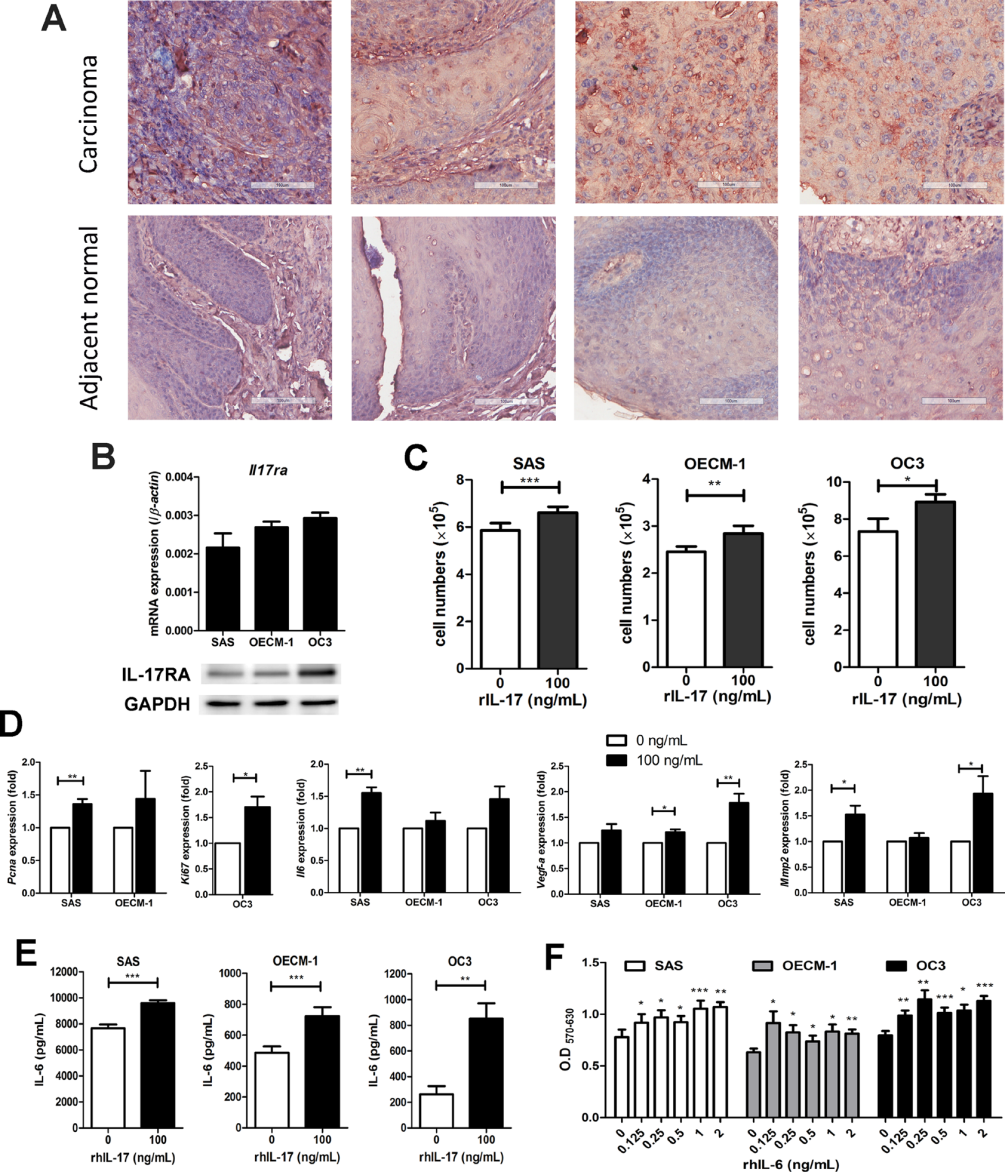
IL-17 induces the proliferation of oral squamous carcinoma cells (OSCC) proliferation through the production of IL-6 (**A**) The increased expression of IL-17RA was analyzed by immunohistochemistry in a tissue array of human oral squamous cell cancer and adjacent normal tongue tissues (200x magnification). (**B**) IL-17RA expression in OSCC cell lines (SAS, OECM-1 and OC3) was analyzed by qRT-PCR assay (upper panel) and western blot analysis (lower panel). (**C**) OSCC cell lines were then treated with 100 ng/mL rhIL-17 for 48 h and the proliferation rates analyzed by cell counting using trypan blue exclusion. (**D**) The expression levels of *Pcna*, *Ki67, Il-6*, *Vegf-a* and *Mmp2* from IL-17-stimulated OSCC cell lines were measured by qRT-PCR and graphed as relative fold changes from untreated cells. (**E**) The OSCC culture supernatants were analyzed for protein levels of IL-6 after treatment with rhIL-17 for 48 h using ELISA. (**F**) OSCC cells were treated with or without different concentrations of rhIL-6 (0.125-2 ng/mL) for 48 h, and cell proliferation was determined by MTT assay. All results represent the mean ± SEM of at last three independent experiments.

Previous reports have suggested that IL-17 signals through interactions with transmembrane receptors composed of IL-17RA and IL-17RC heterodimers induce a broad tissue response at the site of inflammation [12] and increase pro-tumor activity on tumor cells [18, 28]. In our previous study, high levels of IL-17 mRNA have been detected in malignant tissues of HNC but not in normal tissues [29]. Therefore, we aimed to validate the expression of IL-17RA in human oral squamous cell carcinomas (OSCC) and further investigate the potential biological impact of IL-17 on HNC development. From the IHC results, we observed an increase in IL-17RA expression in OSCC tissues compared to adjacent normal tissues (Figure 3A). The expression of IL-17RA was obviously detected in three human OSCC cell lines (SAS, OECM-1, and OC3) at both the mRNA and protein levels (Figure 3B). These data show that IL-17RA expression was up-regulated in OSCC tumor cells and tissues.

To evaluate the impact of IL-17 on OSCC cells via IL-17RA signaling pathways, we stimulated OSCC cell lines with exogenous IL-17 to examine biological function changes *in vitro*. Compared to the untreated groups, the three OSCC cell lines had a significant increase in cell growth following the treatment with 100 ng/mL rhIL-17 for 48 h (Figure 3C). Furthermore, we performed qRT-PCR on IL-17-treated OSCC cells to examine the mRNA levels of inflammatory and pro-angiogenic factors. Our results showed that IL-17 selectively up-regulated gene expression in all three OSCC cell lines. SAS cells expressed higher levels of *Il17ra*, *Pcna*, *Il6,* and *Mmp2*; the enhanced expression of *Il17ra* and *Vegf-a* was also found in OECM-1; OC3 cells showed approximately 1.5-fold greater expression of *Ki67* and the proangiogenic genes *Vegf-a* and *Mmp2* (Figure 3D and Supplementary Figure 2A).

In addition, we found significantly elevated IL-6 production in the supernatants from all three IL-17-treated OSCC cell lines compared to the untreated groups (Figure 3E). Meanwhile, cell proliferation of the OSCC cell lines was significantly enhanced in the cells treated with rhIL-6 (Figure 3F). Moreover, in response to IL-17, NF-κB expression was activated (Supplementary Figure 2B), and the phosphorylation of MAPKs p38, JNK, ERK1/2, and STAT3 were increased in SAS cells (Supplementary Figure 2C). These results suggested that IL-17 has the potential to mediate IL-6 induction in OSCC cells and promote tumor cells proliferation.

### The high prevalence of IL-17-expressing T cells was positively correlated with VEGF-A production in PBMCs in HNC and facilitated OSCC cell proliferation

**Figure 4:**
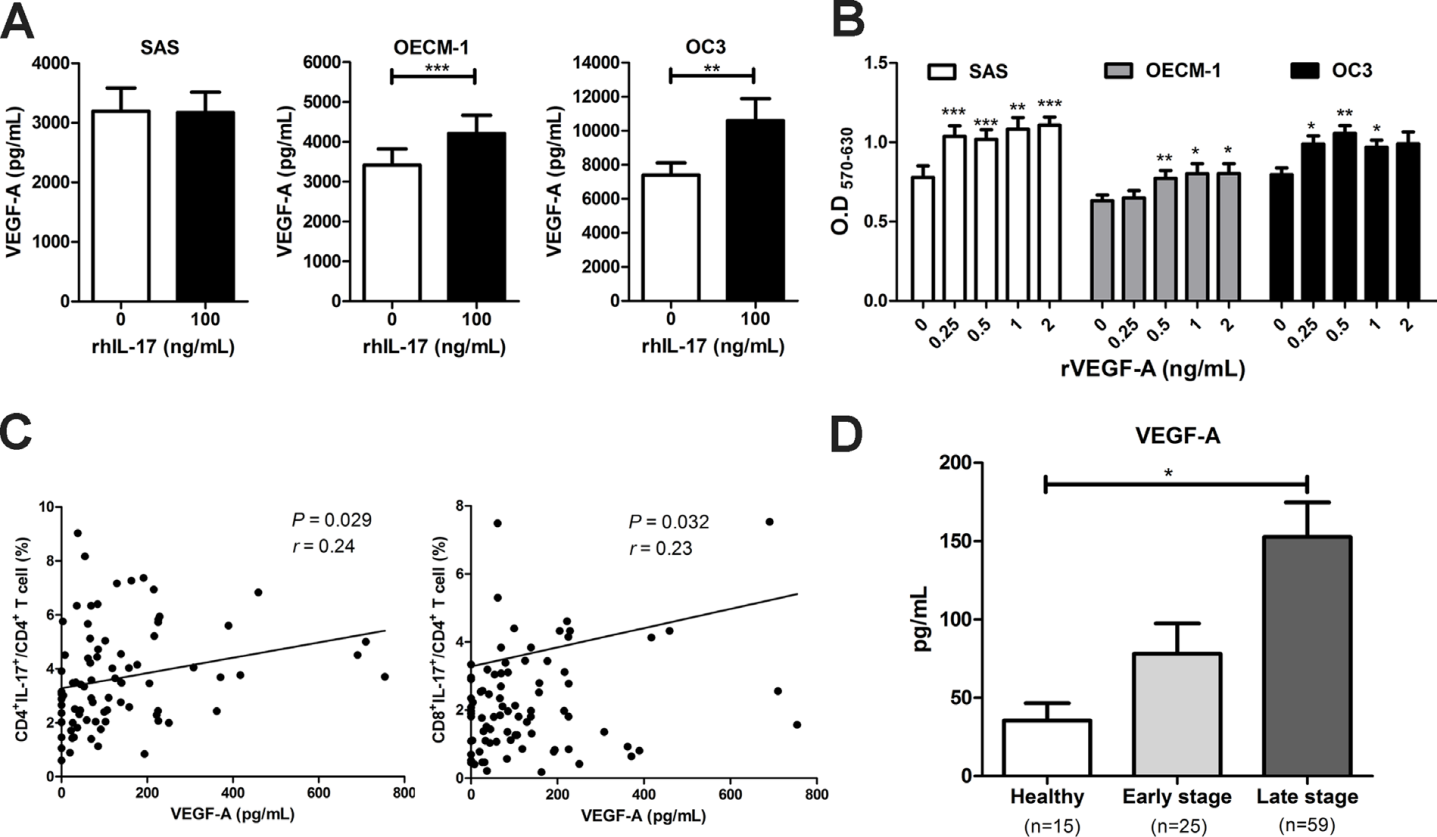
The high expression of IL-17-producing T cells was positively correlated with VEGF-A production in HNC and to facilitate OSCC cells proliferation (**A**) OSCC cells were cultured in the presence of 100 ng/mL rhIL-17 for 48 h and the VEGF-A content from culture supernatants were analyzed for by ELISA. (**B**) OSCC cells were treated with or without different concentrations of recombinant VEGF-A (0.25-2 ng/mL) for 48 h, and cell proliferation was determined by MTT assay. (**C**) Correlations between the level of IL-17-producing T cells (Th17 and Tc17) and plasma VEGF-A in HNC patients (*n* = 84) were analyzed by Pearson correlation test. (**D**) The production of VEGF-A in plasma from HNC patients (early and late stage) and healthy donors were analyzed by ELISA. Data are representatives of at least three experiments and shown as the mean ± SEM.

It has been reported that VEGF-A is one of the IL-17-induced proangiogenic factors that promote angiogenesis and tumor growth [18, 22]. In the present study, we found that although VEGF-A production was not affected by the IL-17 treatment of SAS cells, the IL-17 treatment significantly induced VEGF-A mRNA expression and protein production in the OSCC cell lines OECM-1 and OC3 cells (Figure 4A). The presence of recombinant VEGF-A (0.25 – 2 ng/mL) increased proliferation in a dose-dependent manner *in vitro* in the OSCC cell lines (Figure 4B). Moreover, the neutralizing antibodies were added to IL-17-treated OSCC cells to investigate whether IL-17 achieves its tumorigenic effects by IL-6 or VEGF-A-dependent pathways. The results showed that the treatment with neutralizing IL-6 and VEGF-A by using blocking mAb significantly reduced IL-17-induced cell proliferation of OECM-1 cells. The IL-17-treated SAS cells with neutralizing IL-6 also indicated a similar result of inhibiting cell proliferation (Supplementary Figure 1).

Furthermore, we examined the correlation between the plasma levels of VEGF-A and the frequency of peripheral IL-17-expressing T cells in patients with HNC. We found that plasma VEGF-A levels were positively associated with the frequencies of Th17 (*r* = 0.24, *P* = 0.029) and Tc17 (*r* = 0.23, *P* = 0.032) cells in PBMCs from HNC patients (Figure 4C). Finally, the level of plasma VEGF-A from patients with early HNC (78.02 ± 19.33 pg/mL) and advanced HNC (152.6 ± 22.12 pg/mL) were higher than in healthy controls (35.36 ± 11.20 pg/mL) (Figure 4D). These results indicated that IL-17 may contribute to the poor prognosis of HNC by inducing the production of VEGF-A in circulation or the tumor microenvironment to promote tumor growth and angiogenesis.

## DISCUSSION

Kryczek et al. were the first to show an increase in IL-17^+^ T cell levels in multiple human and mouse tumors [19]. Since then, clinical investigations have addressed the crucial role of IL-17 in cancer surveillance. Most studies have focused on the pathological associations of tumor-infiltrating IL-17^+^ cells or Th17 cells in the tumor tissue rather than on the cells in peripheral circulation [18, 27, 30, 31]. In the present study, we demonstrated the clinical relevance of peripheral IL-17-expressing cells and IL-17 production in patients’ PBMCs in HNC progression. Importantly, our results imply that the increased frequency of inflammatory IL-17-expressing cells, particularly T cells in the peripheral blood, could serve as potential predictors of the poor prognosis of HNC patients.

To improve tumor control and the long-term survival of cancer patients, a better understanding of the molecular basis of the disease is necessary. Previous studies have suggested that the pathological tumor depth and the invasive front tumor grading of HNC are important predictors of clinical outcome [32]. Multiple molecular signatures, such as EGFR and p53, are effective diagnostic tools for HNC. Some of these molecules can be used to determine tumor progression and monitor disease status while others can be used to predict the response to therapy, including survival or recurrence [33]. However, compared to detecting molecular signatures at tumor margins by biopsy, risk factors detected from peripheral circulation, including circulating protein or cellular factors (inflammatory cytokines, circulating tumor cells and immune cell subsets), are more easily accessible and also can provide important information regarding cancer progression, prognostic and therapeutic responses. In fact, such soluble factors not only in serum and plasma but also in other body fluid such as urine, saliva and etc. could be served as the biomarkers. Indeed, the other researchers had assessed the presence of salivary cytokines (e.g., IFN-γ, IL-1β, IL-6, IL-8, IL-10, TNF-α, VEGF) in patients with head and neck cancer (HNC). However, no differences were observed in the salivary cytokine levels between the HNC patients and controls [34].

Herein, the high prevalence of peripheral IL-17-expressing cells in HNC patients was positively associated with the tumor stage, and the higher frequency of peripheral Th17 cells was in accordance with studies on other human tumors [16, 17, 35, 36]. Additionally, an increase in Tc17 cell and/or IL-17^+^ cell frequency has also been detected in peripheral blood of HNC patients. These results suggest that IL-17^+^ cells, including Th17 and Tc17 cells, may be involved in HNC pathogenesis. Recent reports indicated that the higher proportion of peripheral Th17 cells in gastric cancer and hepatocellular carcinoma patients [37, 38] and accumulated IL-17^+^ cells in tumor tissues of larynx carcinoma are correlated with a poor prognosis [39]. Therefore, we examined the prognostic value of measuring IL-17-expressing cell levels in PBMCs of HNC patients. Indeed, we found that the increased levels of peripheral IL-17^+^ cells, IL-17-expressing T cells and their IL-17 production were associated with a worse clinical outcome for HNC patients. Kaplan-Meier analysis revealed that enrichment of Th17, Tc17 and IL-17^+^ cells in the peripheral blood were linked to poor overall survival in HNC patients, indicating that peripheral IL-17-expressing cells levels could potentially serve as a prognostic marker for HNC. Additionally, the multivariate Cox proportional hazards assessment suggested these variables were not independent predictor of overall survival of HNC patients. However, patients with high levels of peripheral IL-17-expressing cells should be closely monitored. Nevertheless, some conflicting research has demonstrated that the presence of tumor-infiltrating IL-17^+^ cells or peripheral Th17 cells is correlated with a favorable outcome and enhanced survival [17, 40]. The associations between IL-17, IL-17-expressing cells, tumorigenesis, and patient prognoses still have no definite conclusion and appear to depend on the cancer type and tissue type (serum, paraffin-embedded tissue, peripheral blood and tumor-associated fluids) used in the study [41, 42]. The multifactorial roles of IL-17 in tumor immunity also depend on the immunogenicity of the tumor, the immune status of the host and the phase of the disease. In the early stage of cancer development, IL-17 may exert anti-tumor activity through different mechanisms, involving IFN-γ dependent manner and stimulating chemokines releasing from tumor cells to recruit immune cells, potentially leading to an enhanced immune response and generate more tumor-specific CD8+ cytotoxic T-lymphocytes (CTL). However, when the cancer development reaches a chronic late stage, the pro-tumor roles of IL-17 and Th17 cells rely on their capacity to induce angiogenesis, recruit inflammatory and immunosuppressive cells and activate tumor-promoting transcription factors [25, 28–30].

The level of IL-17, the most prominent cytokine secreted by Th17/Tc17 cells, in the culture supernatant of PBMCs was increased in HNC patients following tumor progression. Other cytokines secreted by PBMCs, including TGF-β and IL-21, which promote the differentiation of Th17/Tc17 cells [15], were also increased in HNC patients. These results suggested that the TGF-β and IL-21 secreted by PBMCs might be key cytokines that participate in Th17/Tc17 cells differentiation. Furthermore, the release of cytokines is usually a response to stimuli, such as stress, inflammation, or tumor. Cytokines can function to modulate the phenotypes of immune cells and are important for cancer pathogenesis. Among the cytokines, IL-17 has been shown to mediate tumor progression by promoting inflammation and stimulating angiogenesis in immune cells, vascular endothelial cells and stromal cells [22, 43].

Since the high levels of IL-17 production in PBMCs were consistent with HNC progression and the expression of IL-17RA (the predominant IL-17 receptor) is also increased, we presumed that IL-17 may have a direct effect on tumor cells in HNC to mediate tumor promotion. In this study, we showed that IL-17 directly promoted tumor proliferation in OSCC cell lines and augmented the secretion of various pro-tumorigenic factors *in vitro*. Consistent with earlier reports that in human cervical and liver cancer IL-17 played a role in tumor-promoting activity by enhancing IL-6 secretion [28, 43], we demonstrated that elevated IL-6 in IL-17-treated OSCC cell lines has the ability to promote OSCC cells proliferation. In support of these findings, our previous research found high levels of IL-17 and IL-6 mRNA expression in human HNC tissues compared with adjacent normal tissues [29]. These results suggested that IL-17 achieves its tumorigenic effects either by a direct effect on the growth of tumor cells or by inducing IL-6 in the tumor microenvironment of HNC.

It has been reported that IL-17 facilitates tumor angiogenesis via the enhancement of proangiogenic factors, including VEGF-A, by tumor cells [18, 22]. In the present study, we showed that different OSCC cell lines appeared to respond differently to IL-17 in terms of target gene expression and VEGF-A production. However, the exogenous supply of VEGF-A promoted OSCC cells proliferation. Notably, we observed a positive correlation between the levels of serum VEGF-A and peripheral Th17 and Tc17 cells in patients with HNC. Research published elsewhere has shown that VEGF-A expression was significantly up-regulated in OSCC tissues in comparison with controls [44]. Our observations are similar in that the higher levels of plasma VEGF-A in HNC patients are positively correlated with tumor progression. These findings support the notion that IL-17 may promote human HNC tumorigenesis via VEGF-A or IL-6-dependent pathways and may represent an underlying mechanism of poor HNC patient outcome.

In conclusion, our study demonstrated that the increase in IL-17-expressing T cells with higher production of IL-17 in the peripheral circulation of HNC patients was associated with tumor progression and patient survival. These findings imply that peripheral IL-17-expressing cells can potentially be an unfavorable prognostic indicator for overall survival of HNC and also support the notion that IL-17 may have a tumor-promoting role. Furthermore, the evaluation of peripheral IL-17-expressing cells combined with other risk factors in HNC patients might provide better diagnostic capabilities and therapeutic procedures and help monitor HNC disease progression.

## MATERIALS AND METHODS

### Patients and blood sample collection

From 2008 to 2013, we enrolled 120 patients in different stages of HNC, who received treatment at Chang Gung Memorial Hospital, Linkou Medical Center (Taoyuan, Taiwan) in this study. We also enrolled 24 healthy volunteers without a medical history of malignant disease during the same period. Peripheral blood samples were first collected from patients before they received surgical or radiotherapeutic treatment. The disease stage of each patient was classified according to the 2002 American Joint Committee on Cancer (AJCC) staging criteria. Patients were divided into the following two groups: those in the early stages (I and II) and those in the advanced stages (III and IV). The characteristics of the study subjects are summarized in Table 1. All patients received follow-up examinations for at least 60 months after treatment or until death. Overall survival was defined as the interval between the date of treatment and date of death or the last known follow-up. This study was approved by the local ethics committee of Chang Gung Memorial Hospital, and written informed consent was obtained from all individuals.

### Flow cytometry analysis

The standard technique of Ficoll–Hypaque density centrifugation of heparinized blood was used to obtain peripheral blood mononuclear cells (PBMCs) from the study subjects. PBMCs (4 × 10^6^) were cultured in RPMI 1640 supplemented with 100 U/ml penicillin, 100 mg/ml streptomycin, 50 mM β-mercaptoethanol, and 5% fetal bovine serum (FBS, GE Healthcare HyClone™, Utah, USA) in 24-well plates and stimulated with 50 ng/mL phorbol 12-myristate 13-acetate and 750 ng/mL ionomycin (Sigma–Aldrich, St. Louis, MO, USA) [17] for one hour before the addition of 1% GolgiPlug (BD Sciences, San Jose, CA, USA). After 4 hours of culture, PBMCs were harvested and stained with FITC anti-human CD4 or CD8, fixed and permeabilized with a fixation/permeabilization reagent, and finally stained with Phycoerythrin (PE) anti-human IL-17A (eBioscience, San Diego, CA, USA). Samples were run on BD FACSCalibur, and FCS ExpressV3 Software (BD Biosciences) was used for the analysis.

### Immunohistochemistry and assessment

Immunohistochemical staining for human IL-17RA was performed using a human oral squamous cell cancer tissue array with adjacent normal tongue tissue (US Biomax Inc., cat. OR601a). For deparaffinization, rehydration and antigen retrieval, the sections were immersed in Trilogy™ solution (Sigma–Aldrich) and autoclaved at 120°C for 20 min. The endogenous peroxide activity was then eliminated with 3% hydrogen peroxide for 10 min. The sections were incubated with primary antibody anti-IL-17R (1:50 dilution, clone G-9, Santa Cruz, Dallas, TX, USA), and a biotinylated goat anti-mouse IgG (Jackson ImmunoResearch, West Grove, PA, USA) was used as a secondary antibody. Diaminobenzidine (Dako, Glostrup, Denmark) was used for visualization, along with counterstaining with hematoxylin for 90 sec, followed by dehydration in alcohol and finally mounted with Assistent HistoKitt mounting medium (Hecht Karl, Germany).

### Quantitative reverse transcription-polymerase chain reaction (qRT-PCR)

Total RNA was extracted from cells using TRIzol (Invitrogen) according to the manufacturer’s instructions. RNA was used to synthesize cDNA using iScript™ cDNA Synthesis Kit, and the gene expression analysis was performed using quantitative real-time PCR using iQ™ SYBR Green Supermix and normalized to β-actin. Each reaction was run in duplicate on the CFX connect real-time PCR detection system (Bio-Rad, Hercules, CA, USA). Primer sequences for the quantitative RT-PCR are listed in Supplementary Table 4.

### Cell lines and culture

SAS cells were maintained in Dulbecco’s Modified Eagle’s medium (DMEM) supplemented with 10% FBS. Oral carcinoma 3 cell lines (OC3) were grown in a 1:2 mixture of DMEM and Keratinocyte-SFM medium (Life Technologies, Carlsbad, CA, USA) supplemented with 10% FBS. Oral epidermoid carcinoma Meng-1 (OECM-1) cells were maintained in RPMI-1640 containing 10% FBS. All cell lines were cultured in a humidified atmosphere of 5% CO_2_ at 37°C. The SAS and OC3 cell lines were kindly provided by Professor Yu-Sun Chang, and the OECM-1 cell line was provided by Professor Ching-Ping Tseng (Graduate Institute of Biomedical Science, Chang Gung University, Taiwan). For the OSCC cells proliferation assay, the OSCC cell lines were seeded in 6-well plates supplemented with recombinant human IL-17 (rhIL-17) or in 96-well plates with recombinant human IL-6 (rhIL-6) or recombinant VEGF-A (Peprotech, Rocky Hill, NJ, USA) for 24–48 h, and the proliferation rates were analyzed by cell counting using trypan blue or 3-(4,5-dimethylthiazol-2-yl)-2,5-diphenyltrazolium bromide (MTT) assay. To determine the effects of IL-6 or VEGF-A on IL-17 treated OSCC cells, the neutralizing antibodies were utilized, including rat anti-IL-6 mAb (Cat. No. 50110, BioLegend, San Diego, CA, USA) and mouse anti-VEGF mAb (MAB293, R&D Systems, Minneapolis, MN, USA). The isotype antibodies were also included as controls.

### Measurement of cytokines

The supernatants of the PBMCs were harvested, and concentrations of IL-17, IL-21, TGF-β (eBioscience) and plasma VEGF-A (R&D Systems) were measured using commercially available ELISA kits. IL-1β, IL-6, and other inflammatory mediators were quantified using human inflammation CBA kits and human chemokine CBA kits (BD Biosciences) following the manufacturer’s instructions. Cell-free supernatants from human oral squamous cell lines treated with or without IL-17 (100 ng/mL) were collected to measure the concentrations of VEGF-A and IL-6 (eBioscience) by ELISA.

### Statistical analysis

The statistical analysis was performed using GraphPad Prism 5.0 (San Diego, CA, USA) and SPSS 12.0 software (SPSS, Chicago, IL, USA). Data are presented as the means ± SEM. Differences between the groups were assessed using *t-*tests. Kaplan–Meier curves, reflecting overall survival rates, and statistical significance was calculated according to the log-rank test. The correlation analysis between cells producing IL-17 and those producing VEGF-A was determined by the Pearson correlation test. All tests were two-tailed, and *P* < 0.05 was considered statistically significant (^*^P < 0.05, ^**^P < 0.01, ^***^P < 0.001).

## SUPPLEMENTARY MATERIALS FIGURES AND TABLES


