# Supplementary material for: Interleukin 17 and peripheral IL-17-expressing T cells are negatively correlated with the overall survival of head and neck cancer patients
Source: Oncotarget. 2018 Jan 3;9(11):9825–37. doi: 10.18632/oncotarget.23934 (PMC5839404; doi:10.18632/oncotarget.23934)
Supplement: Supplementary file 1 [file oncotarget-09-9825-s001.pdf]

Interleukin 17 and peripheral IL-17-expressing T cells are negatively correlated with the overall survival of head and neck cancer patients

SUPPLEMENTARY MATERIALS

Supplementary Table 1: Associations of IL-17-expressing T cells in PBMC with clinical and pathologic features for HNC patients

Characteristics	No. of HNC (%)	CD4+IL17+/CD4 (%)			CD8+IL17+/CD8 (%)		
		mean	(min-max)	p^c	mean	(min-max)	p
Age (years)							
50	58 (48.3)	3.45	(0.60–9.03)	0.907	2.40	(0.41–8.57)	0.669
50	62 (51.7)	3.48	(0.84–7.37)		2.28	(0.18–7.49)	
Gender							
Male	99 (82.5)	3.63	(0.60–9.03)	0.019	2.44	(0.18–8.57)	0.139
Female	21 (17.5)	2.68	(1.06–5.21)		1.87	(0.22–7.53)	
T classification[a,b]							
T1+T2	75 (62.5)	3.15	(0.60–9.03)	0.008	2.13	(0.22–7.53)	0.063
T3+T4	45 (37.5)	3.99	(0.84–8.17)		2.69	(0.18–8.57)	
N classificationa[b]							
N0	45 (37.5)	3.15	(1.06–7.27)	0.115	2.32	(0.18–8.57)	0.921
N+	75 (62.5)	3.65	(0.60–9.03)		2.35	(0.41–7.53)	
Overall stagea[b]							
I+II	44 (36.7)	2.83	(1.06–6.40)	0.002	2.15	(0.22–6.45)	0.320
III+IV	76 (74.3)	3.83	(0.60–9.03)		2.45	(0.18–8.57)	

[a]Pathological T/N classification and stage for oral cavity cancer.
[b]Clinical T/N classification and stage for oral pharyngeal, laryngeal, and hypopharyngeal cancer.
[c]P values were determined by *t* test.

Supplementary Table 2: Levels of inflammatory cytokines produced form PBMC contributing to HNC clinicopathologic status

	TNF	IL-10	IFN-γ	IP-10	MIG	MCP1
Healthy donor	2129 ± 569.8	55.77 ± 16.14	6134 ± 1763	25.64 ± 11.49	30.08 ± 11.02	115.9 ± 58.75
HNC	1675 ± 211.4	21.67 ± 3.42**	11473 ± 1971	26.98 ± 6.755	26.26 ± 5.0	422.2 ± 82.92
Early stage (I+II)	1909 ± 354.5	20.7 ± 4.89*	9137 ± 3750	30.16 ± 11.46	30.48 ± 9.22	301.3 ± 105.1
Late stage (III+IV)	1537 ± 264.2	22.24 ± 4.647**	12852 ± 2231	25.1 ± 8.45	23.77 ± 5.85	494.7 ± 115.8

[a]Healthy donor $n = 11$, Early stage $n = 23$, Advanced stage $n = 39$.
[b]Data were presented as means ± SEM (pg/mL).
[c]P value reflects t test analysis of cytokine values from HNC patients compared with healthy donors.
$^{*}P < 0.05$, $^{**}P < 0.01$, $^{***}P < 0.001$

Supplementary Table 3: Univariate and multivariate analysis of factors associated with overall survival in HNC

Variables	Univariate			Multivariate		
	HR[a]	95% CI	*P* value[b]	HR[b]	95% CI	*P* value[c]
Age (years, > 50 vs. ≤ 50)	1.475	0.728–2.987	0.281			
Gender (female vs. male)	0.624	0.276–1.413	0.258			
T stage (T3+T4 vs. T1+T2)	2.260	1.094–4.670	0.028*	0.929	0.350–2.467	0.882
Lymph node (N+ vs. N0)	2.090	1.002–4.357	0.049*	1.940	0.711–5.291	0.195
TNM Stage (III+IV vs. I+II)	2.418	1.177–4.970	0.016*	1.484	0.432–5.096	0.531
CD4+ IL-17+ cells (high vs. low)	2.095	1.032–4.253	0.041*	1.566	0.711–3.449	0.266
CD8+ IL-17+ cells (high vs. low)	2.568	1.260–5.231	0.009**	1.839	0.782–4.321	0.162
IL-17+ cells (high vs. low)	2.591	1.272–5.279	0.009**	2.010	0.886–4.556	0.095

[a]HR, hazard ratio; CI, confidence interval. HR > 1, risk for death increased; HR < 1, risk for death decreased.
[b]Log-rank test.
[c]Multivariate Cox proportional hazards regression model.
Statistically significant ($P < 0.05$, **$P < 0.01$).

Supplementary Table 4: Primer sequences used for real-time qPCR

Name	Forward primer	Reverse primer
Primers specific for human		
Il17ra	5′-CTTCACCCTGTGGAACGAAT-3′	5′-CTGAAGAAGGGCTGGATCTG-3′
Il6	5′-GAGACATGTAACAAGAGTAA-3′	5′-AGGCAAGTCTCCTCAT-3′
Pcna	5′-GCACTCAAGGACCTCATC-3′	5′-AACTTTCTCCTGGTTTGG-3′
Mmp2	5′-TGGCAAGTACGGCTTCTGTC-3′	5′-TTCTTGTCGCGGTCGTAGTC-3′
Ki67	5′-ACGAGACGCCTGGTTACTATC-3′	5′-GCTCATCAATAACAGACCCATTTAC-3′
Vegf-a	5′-CTTGCCTTGCTGCTCTACC-3′	5′-CACACAGGATGGCTTGAAG-3′

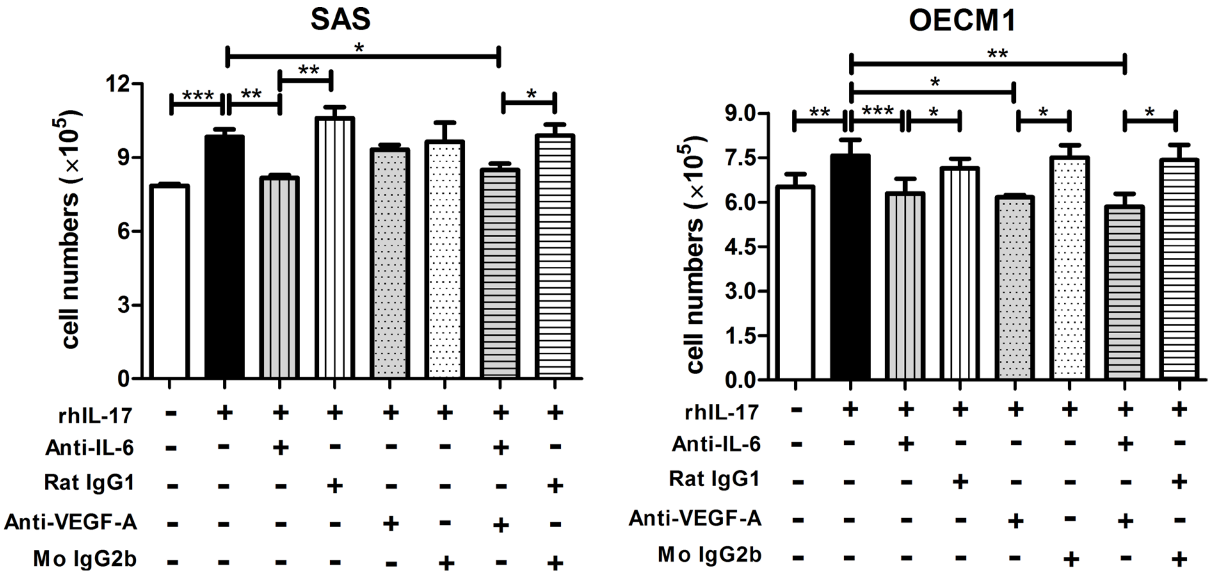

Supplementary Figure 1: Neutralizing IL-6 and VEGF-A by using blocking mAb significantly reduced IL-17-induced cell growth of OSCC cells. The neutralization of endogenous IL-6 or VEGF-A produced by rhIL-17-treated OSCC cells. SAS and OECM-1 cells were treated with 100ng/mL rhIL-17 and either 0.1μg/mL neutralization antibodies (anti-IL-6 and anti-VEGF) or isotype control antibodies (rat IgG1 and mouse IgG2b) for 48 h, and the proliferation rates analyzed by cell counting using trypan blue exclusion. All results represent the mean ± SEM of at last four independent experiments ($^{*}P < 0.05$, $^{**}P < 0.01$, $^{***}P < 0.001$).

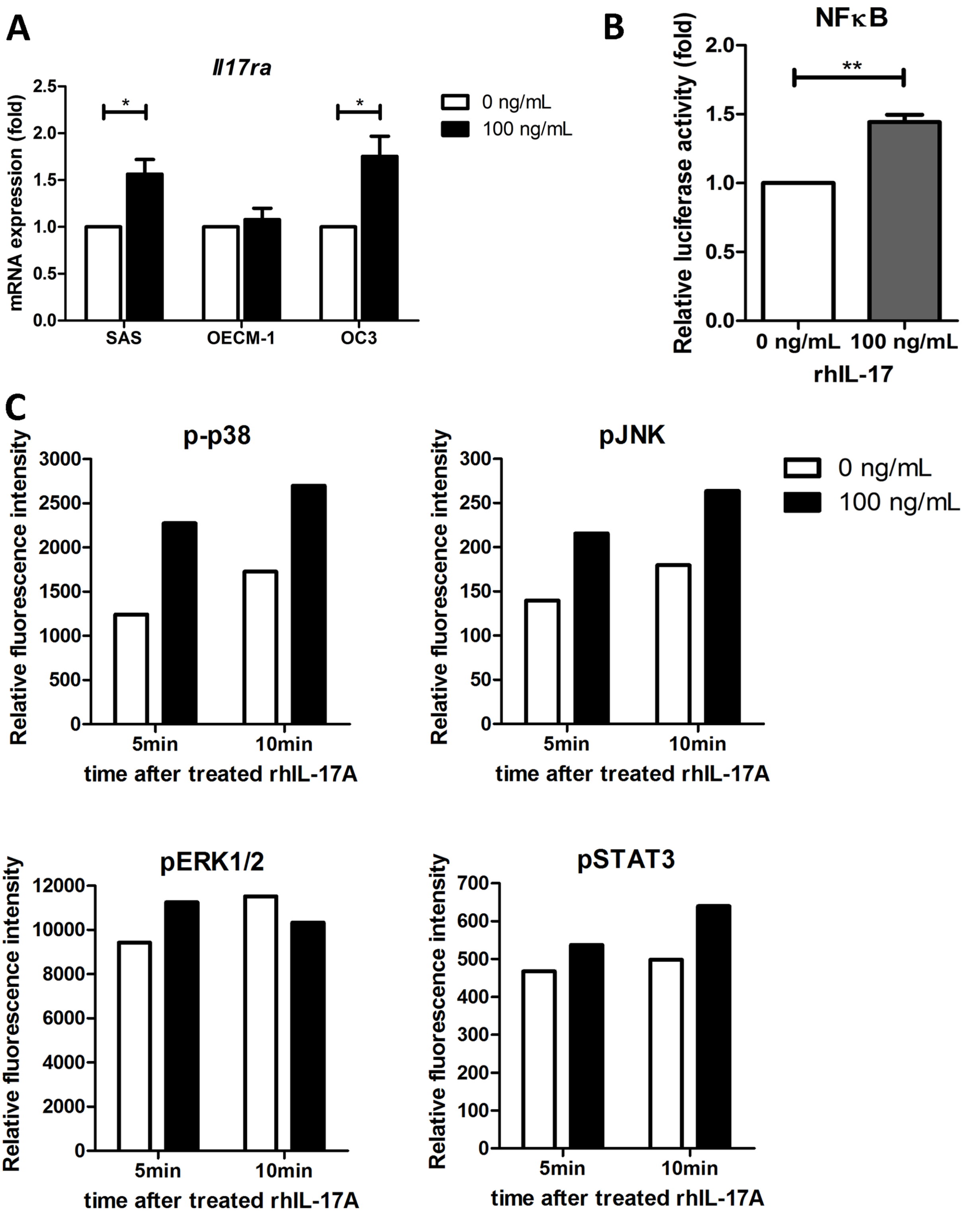

Supplementary Figure 2: Cell signaling analysis of IL-17-stimulated SAS cells. (**A**) The expression levels of *Il-17ra* from IL-17-stimulated OSCC cell lines were measured by qRT-PCR and graphed as relative fold over untreated. (**B**) SAS cell was transfected with NF-kB promoter-luciferase plasmid, cultured in the presence or absence of 100 ng/ml rhIL-17 for 6h, and luciferase activity was measured to assess the NF-kB activity. Data are representatives of five experiments and shown as mean ± SEM. (**C**) Cell signaling analysis of IL-17-stimulated SAS cells for the indicated times. The phosphorylation of protein levels was quantified using cell signaling multiplex assay.

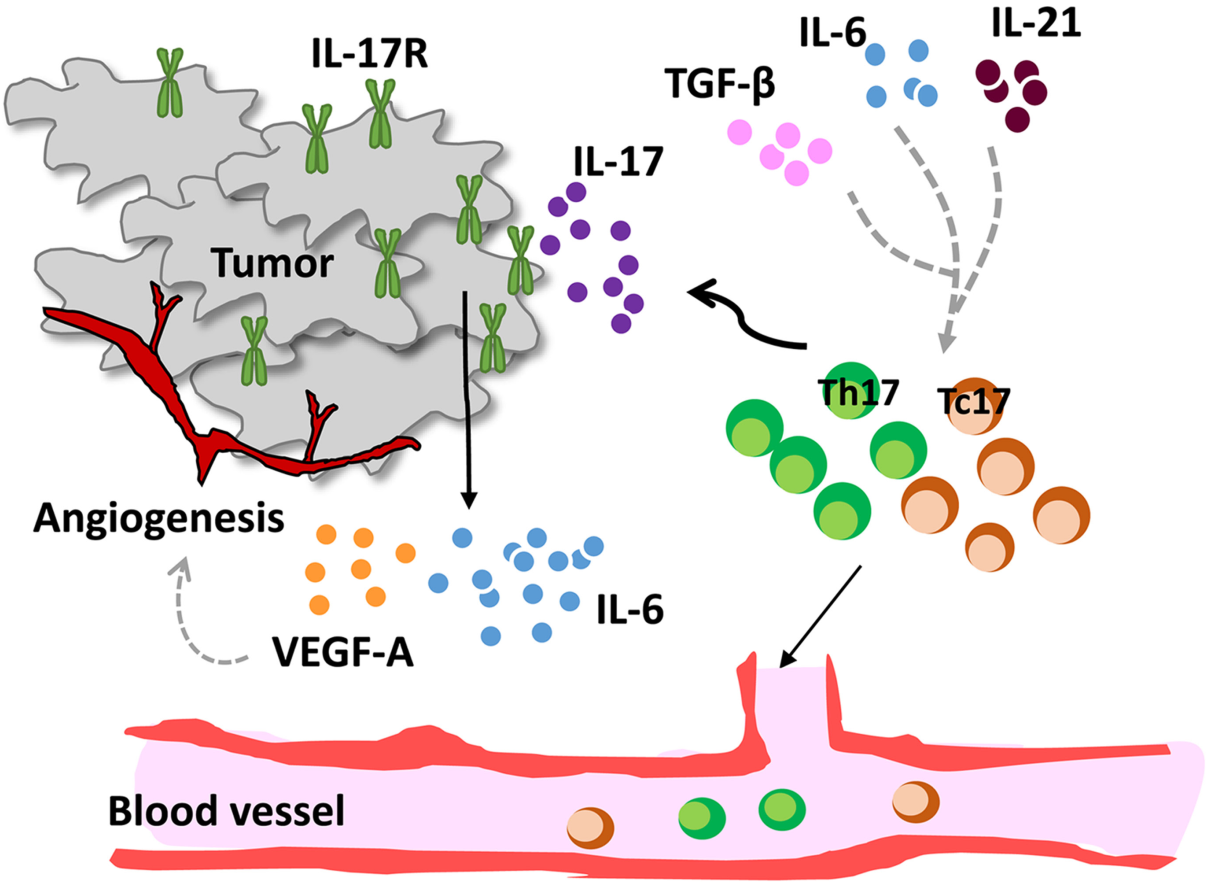

Supplementary Figure 3: The schematic diagram demonstrates the possible mechanisms of IL-17 contributing to HNC progression and of some Th17 and Tc17 releasing into the periphery. As tumors develop and progress, the induced production of TGF-β, IL-21 and/or IL-6 by PBMCs promotes the differentiation of Th17/Tc17 cells, which produce a large quantity of IL-17. In the tumor microenvironment, IL-17 acts on tumor cells to stimulate the expression of IL-6 and VEGF-A, which consequently enhances Th17 differentiation in a positive feedback loop and creates a sustained chronic inflammatory state that favors tumor growth and angiogenesis. As tumor progression, the abundantly elicited IL-17-expressing cells, including Th17 and Tc17 cells, released into circulation and could be found in peripheral blood of patients with HNC.
